# Case Report: Acute keratoconus as the presenting feature in undiagnosed Norrie disease: hypothesis from a novel NDP mutation

**DOI:** 10.3389/fmed.2026.1726644

**Published:** 2026-03-30

**Authors:** Xu Cao, Longhao Kuang, Jiexuan Lv, Shaoyi Mei, Jingjing Su, Yufang Wang, Qing Zhan, Ping Guo

**Affiliations:** 1 Shenzhen Eye Hospital, Shenzhen Eye Medical Center, Southern Medical University, Shenzhen, Guangdong, China; 2 Nanshan College, Guangzhou Medical University, Guangzhou, Guangdong, China

**Keywords:** acute hydrops, keratoconus, NDP gene, Norrie disease, whole-genome sequencing, X-linked recessive

## Abstract

**Background:**

Norrie disease is a rare X-linked recessive disorder characterized by congenital blindness, with approximately one-third of patients developing progressive sensorineural hearing loss and neurodevelopmental abnormalities. While over 200 pathogenic variants in the *NDP* gene have been identified, the phenotypic spectrum continues to expand.

**Case Presentation:**

We report a 33-year-old Chinese male who presented with acute hydrops of the right cornea as the initial clinical manifestation leading to the diagnosis of Norrie disease. Comprehensive ophthalmological examination revealed bilateral keratoconus (right eye with acute Descemet’s membrane rupture), spherophakia, shallow anterior chambers, and characteristic grayish-white fibrovascular masses in the vitreous cavity. Whole-genome sequencing identified a novel hemizygous mutation in *NDP* (NM_000266.4: c.140_144delinsTTTTA), resulting in p. Ser47_Ile48delinsPheLeu. This mutation affects highly conserved residues adjacent to the cysteine knot domain critical for Norrin protein function. The proband’s mother was a heterozygous carrier with normal phenotype, confirming X-linked recessive inheritance. In silico analysis using multiple prediction algorithms uniformly indicated pathogenicity.

**Conclusion:**

This represents, to our knowledge, the first report of Norrie disease presenting with acute keratoconus (hydrops) as the initial diagnostic manifestation in an undiagnosed adult. The novel p. Ser47_Ile48delinsPheLeu mutation expands both the mutational spectrum of *NDP* and the phenotypic presentation of Norrie disease. These findings emphasize the importance of genetic testing in patients with complex anterior segment abnormalities and provide crucial information for genetic counseling and clinical management.

## Background

X-linked vitreoretinopathies represent a heterogeneous group of inherited retinal disorders with significant phenotypic overlap and genetic heterogeneity (1). Among these, Norrie disease (ND; MIM: 310600) stands out as a severe form affecting multiple organ systems, with an estimated incidence of 1:100,000 live births (2). The condition is characterized by congenital blindness due to bilateral retinal dysplasia, often accompanied by progressive sensorineural hearing loss in approximately 30–50% of cases and neurodevelopmental delay in one-third of affected individuals (3, 4).

The causative gene, *NDP* (Norrie disease pseudoglioma), maps to chromosome Xp11.3 and comprises three exons encoding a 133-amino acid secreted protein, Norrin (3). This cysteine-rich protein functions as an atypical Wnt ligand, specifically activating the canonical Wnt/*β*-catenin signaling pathway through high-affinity binding to the Frizzled-4 (FZD4) receptor and its co-receptors LRP5/6 and TSPAN12 (5–7). This signaling cascade plays a crucial role in retinal vascular development, blood-retinal barrier formation, and inner ear development (6).

To date, more than 200 pathogenic variants in *NDP* have been documented, including missense, nonsense, frameshift, and deletion mutations (8). Despite this extensive mutational catalogue, genotype–phenotype correlations remain incompletely understood, and novel clinical presentations continue to emerge. Here, we report a Chinese family with Norrie disease caused by a novel *NDP* mutation, uniquely presenting with acute keratoconus as the initial clinical manifestation, thereby expanding the recognized phenotypic spectrum of this disorder.

## Case presentation

### Clinical history and examination

A 33-year-old Chinese male presented to our emergency department with a two-week history of severe right eye pain, photophobia, and sudden visual deterioration. The patient reported a lifelong history of poor vision in both eyes but had not sought previous ophthalmological evaluation. There was no history of atopy, eye rubbing, or connective tissue disorders. Family history was unremarkable for visual impairment or hearing loss. The patient denied any subjective hearing impairment. Formal audiometric testing was not performed during this presentation, as the primary concern was ocular. No prior diagnosis or history of progressive hearing loss was reported. No history of developmental delay, intellectual disability, behavioral abnormalities, autism spectrum features, or other neurodevelopmental concerns was reported by the patient or elicited from family members. The patient lives independently and reports normal cognitive and daily functioning.

On examination, best-corrected visual acuity (BCVA) was finger counting at 30 cm in the right eye and 0.03 (Snellen equivalent: 20/600) in the left eye. Both eyes exhibited horizontal nystagmus and 15° esotropia. Detailed ophthalmological findings are summarized in Table 1.

**Table 1:** Clinical Features of the Proband.

**Parameter**	**Right Eye**	**Left Eye**
Visual Acuity (BCVA)	FC at 30 cm	0.03 (20/600)
Alignment	15° esotropia	15° esotropia
Nystagmus	Horizontal	Horizontal
Cornea	Acute hydrops, edema, DMD rupture	Clear
Corneal Thickness (μm)	360	554
Keratometry (D)	K1: 84.3, K2: 92.8, Km: 112.1	K1: 43.5, K2: 45.3, Km: 49.4
Anterior Chamber	>4 CT (central)	1.5 CT (central), 0.25 CT (peripheral)
Lens	Not visible	Spherical, mild opacity
Vitreous	Not visible	Fibrovascular mass
IOP (mmHg)	14	15

BCVA: Best-corrected visual acuity; FC: Finger counting; DMD: Descemet’s membrane; CT: Corneal thickness; D: Diopters; IOP: Intraocular pressure.

**Figure 1:**
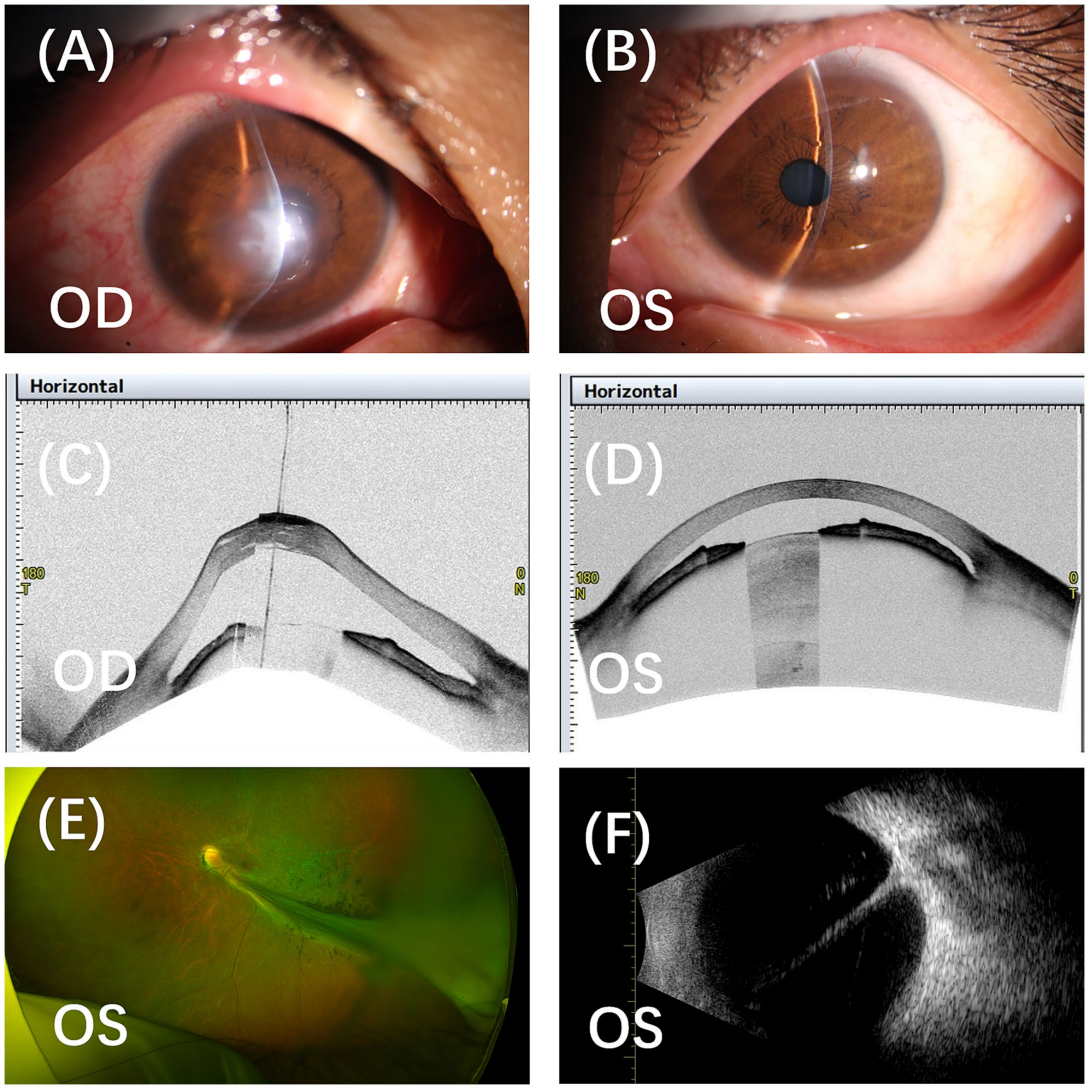
Clinical imaging of the proband’s ocular manifestations. **(A)** Anterior segment photograph of the right eye showing central corneal protrusion, stromal edema, opacity, and Descemet’s membrane rupture characteristic of acute hydrops. **(B)** Anterior segment photograph of the left eye demonstrating clear cornea with shallow anterior chamber (central depth 1.5 CT, peripheral 0.25 CT) and mild lens opacity. **(C)** Anterior segment OCT of the right eye revealing severe conical protrusion, extensive stromal edema, intrastromal clefts (arrows), and Descemet’s membrane rupture (arrowhead). **(D)** Anterior segment OCT of the left eye revealing normal corneal architecture. **(E)** Scanning laser ophthalmoscopy of the left eye revealing a grayish-white fibrovascular mass adherent to the retina, characteristic of Norrie disease. **(F)** B-scan ultrasonography of the left eye demonstrating hyperechoic vitreous opacities with membranous attachments to the optic disc. CT, corneal thickness.

The right eye demonstrated classic signs of acute corneal hydrops with central corneal protrusion, stromal edema, and Descemet’s membrane rupture visible on slit-lamp biomicroscopy (Figure 1A). Munson’s sign was positive. The anterior chamber depth exceeded 4 corneal thicknesses centrally, consistent with severe keratoconus. Posterior segment details were obscured by corneal opacity.

The left eye showed a clear cornea with a central anterior chamber depth of 1.5 corneal thicknesses and peripheral shallowing to 0.25 corneal thicknesses (Figure 1B). A spherical, mildly opacified lens was noted.

### Imaging studies

**Figure 2:**
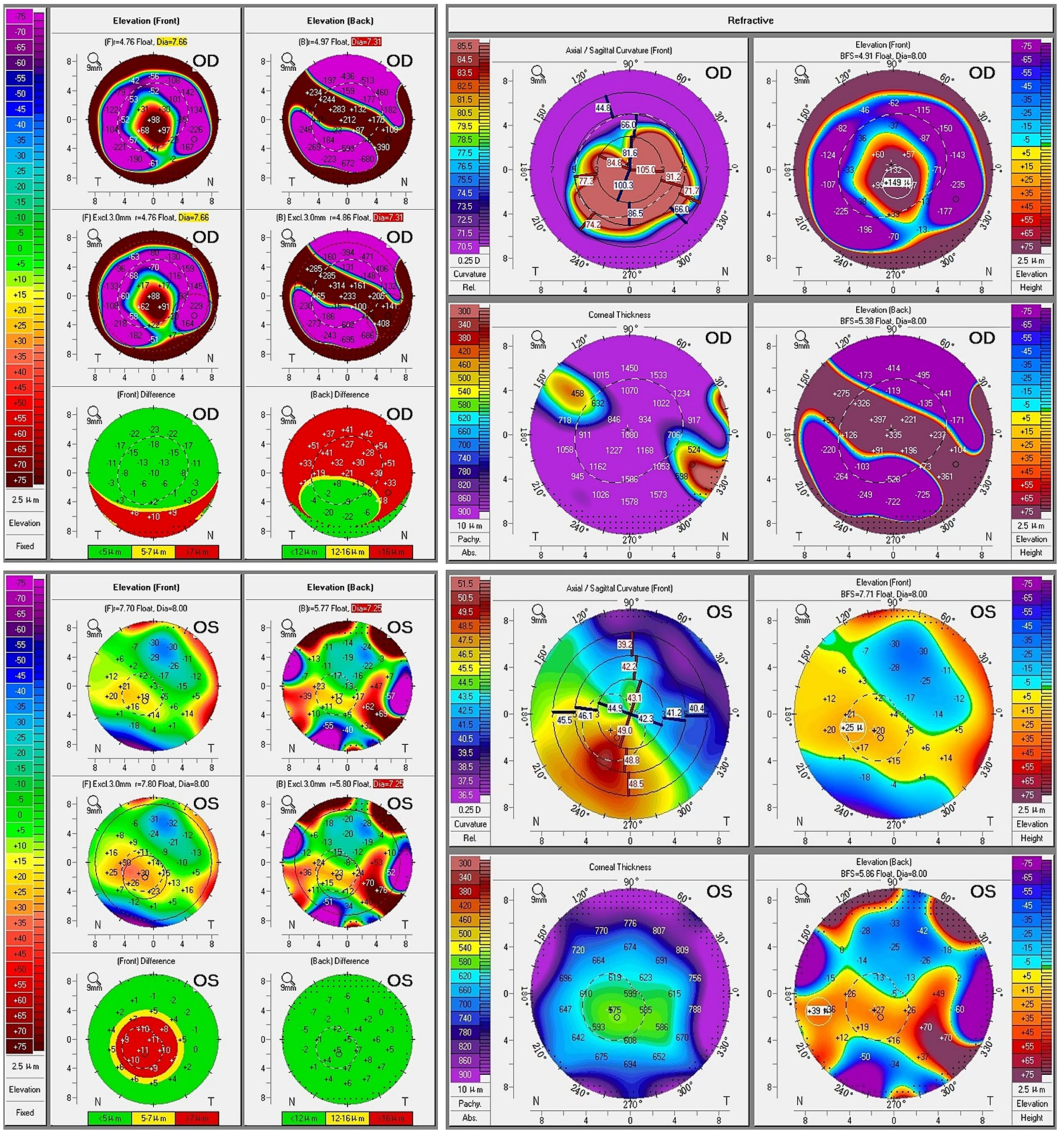
Comprehensive Pentacam corneal topography and tomography analysis of both eyes. Upper panel (OD–right eye): Shows severe keratoconus with acute hydrops. Elevation maps (front and back) demonstrate extreme anterior protrusion with maximum elevation of >76 μm (front, shown in red/white) and severe posterior depression. Pachymetry map reveals central thinning (360 μm). Sagittal curvature maps show extreme steepening with Kmax exceeding 85 diopters. The Belin/Ambrósio Enhanced Ectasia Display (right side) shows all parameters severely abnormal (red), with final D value of 26.91. Front and back difference maps indicate significant deviation from reference surface (>57 μm front difference, >121 μm back difference). Lower panel (OS–left eye): Demonstrates moderate keratoconus. Elevation maps show anterior elevation up to +26 μm and posterior elevation changes. Pachymetry map shows relative central thinning (554 μm). Sagittal curvature maps reveal inferior steepening pattern typical of keratoconus with Kmax of 52.7 diopters. The Belin/Ambrósio display shows multiple abnormal parameters with final *D*-value of 5.86. Difference maps confirm ectatic changes with focal elevation differences. Color scales represent elevation in micrometers (μm) for elevation maps, corneal power in diopters (D) for curvature maps, and thickness in micrometers for pachymetry maps. OD, right eye; OS, left eye.

Pentacam corneal topography (Oculus, Wetzlar, Germany) demonstrated severe bilateral keratoconus with markedly elevated curvature values (Figure 2). The right eye showed extreme steepening with K1/K2/Km values of 84.3/92.8/112.1 diopters and central corneal thickness of 360 μm. The left eye exhibited K1/K2/Km values of 43.5/45.3/49.4 diopters with central thickness of 554 μm. Belin/Ambrósio Enhanced Ectasia Display showed multiple parameters exceeding normal ranges bilaterally.

Anterior segment optical coherence tomography (AS-OCT; Visante, Carl Zeiss Meditec) of the right eye revealed pronounced conical protrusion, extensive stromal edema, intrastromal clefts, and a clear break in Descemet’s membrane (Figure 1C). The left cornea appeared structurally intact (Figure 1D). Biometry (IOLMaster 700, Carl Zeiss Meditec) of the left eye measured anterior chamber depth of 1.52 mm and lens thickness of 5.95 mm, confirming spherophakia.

Fundoscopy under pharmacological mydriasis of the left eye revealed a large grayish-white fibrovascular mass in the vitreous cavity, characteristic of Norrie disease (Figure 1E). B-scan ultrasonography (Aviso, Quantel Medical) demonstrated bilateral vitreous opacities with positive aftermovement and membranous echoes adherent to the optic disc (Figure 1F). The right eye additionally showed a suspected area of retinal detachment along the nasal periphery.

### Family evaluation

**Figure 3:**
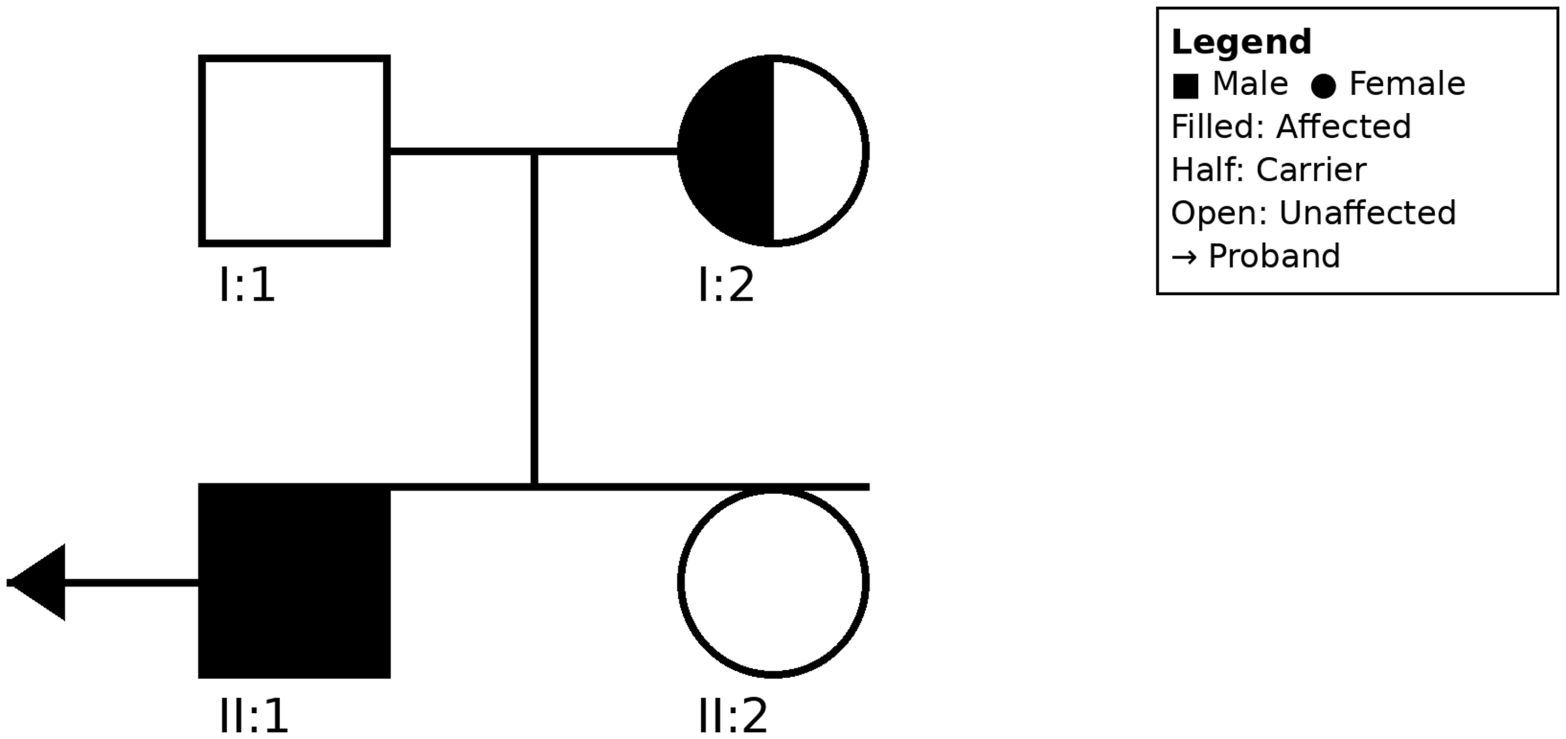
Pedigree of the Chinese family with Norrie disease. Squares represent males; circles represent females. Filled symbol indicates affected individual (proband, II:1). Half-filled symbol indicates heterozygous carrier (I:2). Arrow indicates the proband. The novel *NDP* mutation (c.140_144delinsTTTTA) segregates with the disease phenotype in an X-linked recessive pattern.

Comprehensive ophthalmological examination of the proband’s parents and sister revealed no abnormalities. The family pedigree is illustrated in Figure 3.

## Methods

### Ethics statement

This study was approved by the Medical Ethics Committee of Shenzhen Eye Hospital and adhered to the tenets of the Declaration of Helsinki. Written informed consent was obtained from all participants for genetic testing and publication of clinical data.

### Genetic analysis

#### DNA extraction and whole-genome sequencing

Peripheral blood samples (5 mL) were collected from family members and 100 ethnically matched control subjects. Genomic DNA was extracted using the QIAamp DNA Blood Mini Kit (Qiagen, Hilden, Germany) following manufacturer’s protocols.

Whole-genome sequencing was performed on the proband’s DNA sample at Beijing Zhide Medical Laboratory. Library preparation was conducted using the NEBNext Ultra DNA Library Prep Kit (New England Biolabs). Sequencing was performed on an Illumina NovaSeq 6,000 platform with 150-bp paired-end reads, achieving mean coverage of 30 × across the genome and >99% coverage at 10 × depth.

#### Bioinformatics analysis

Raw sequencing data were aligned to the human reference genome (GRCh38/hg38) using BWA-MEM (v0.7.17). Variant calling was performed using GATK HaplotypeCaller (v4.2.0) following best practices guidelines. Variants were annotated using ANNOVAR and filtered against population databases including gnomAD, 1,000 Genomes Project, ESP6500, and an in-house database of 500 Chinese controls.

Pathogenicity prediction was assessed using multiple in silico tools: PolyPhen-2 (v2.2.2), SIFT (v6.2.1), MutationTaster2021, CADD (v1.6), and REVEL. Conservation analysis was performed using PhyloP and PhastCons scores across 100 vertebrate species.

#### Variant validation

Candidate variants were validated by Sanger sequencing using primers flanking the mutation site (Table 2). PCR amplification was performed using Q5 High-Fidelity DNA Polymerase (New England Biolabs) with the following conditions: initial denaturation at 94 °C for 3 min; 35 cycles of 94 °C for 40 s, 53 °C for 40 s, and 72 °C for 60 s; final extension at 72 °C for 10 min. Sequencing was performed on an ABI 3730xl DNA Analyzer (Applied Biosystems).

**Table 2:** PCR Primers for NDP Gene Sequencing.

**Primer**	**Sequence (5' to 3')**
NDP-F	AGGCACCACTATGTGGATTCTATC
NDP-R	GCTTGAGGACAGTGCTGAACGA

## Results

### Genetic findings

**Figure 4:**
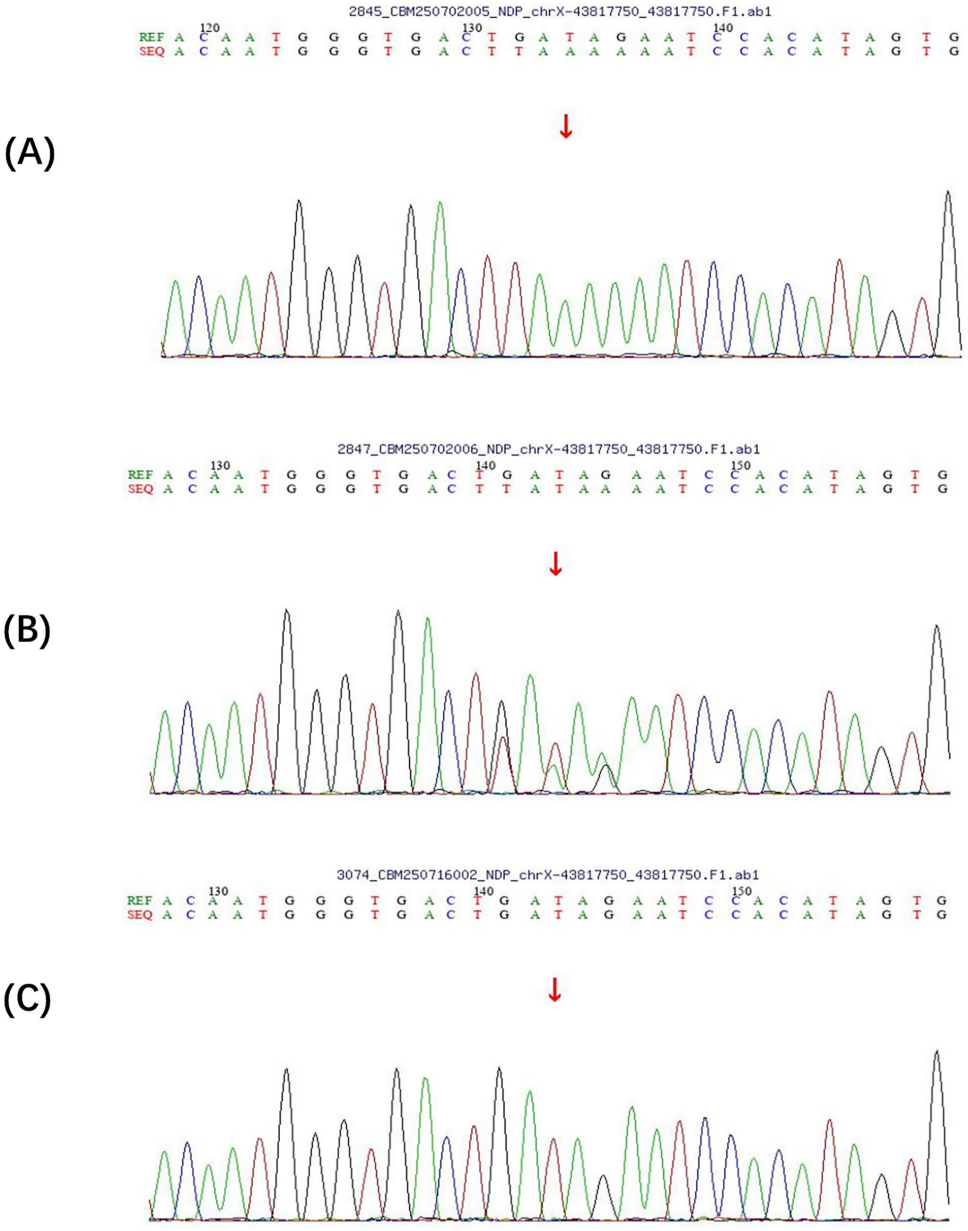
Sanger sequencing validation of the *NDP* gene mutation (c.140_144delinsTTTTA) in the family. **(A)** Hemizygous mutation in the proband showing complete replacement of the wild-type sequence (ACTGA) with mutant sequence (TTTTA) at positions c.140–144, indicated by the red arrow. The chromatogram shows clean, single peaks confirming the hemizygous state. **(B)** Heterozygous carrier status in the proband’s mother displaying overlapping peaks at the mutation site (red arrow), demonstrating the presence of both wild-type (ACTGA) and mutant (TTTTA) alleles characteristic of a carrier female. **(C)** Normal wild-type sequence in an unaffected family member showing the reference sequence ACTGA at positions c.140–144 with clear, single peaks. The mutation results in amino acid changes p. Ser47_Ile48delinsPheLeu. REF: reference sequence; SEQ: sequencing result.

Whole-genome sequencing identified a novel hemizygous variant in the *NDP* gene (NM_000266.4): c.140_144delinsTTTTA, resulting in p. Ser47_Ile48delinsPheLeu (Figure 4A). This complex indel mutation replaces five nucleotides with five different nucleotides, leading to the substitution of two consecutive amino acids in the mature Norrin protein.

The affected residues (Ser47 and Ile48) are located immediately adjacent to the cysteine knot domain (residues 49–131), a critical structural motif essential for Norrin protein stability and receptor binding. Multiple sequence alignment demonstrated complete conservation of these residues across all examined vertebrate species (Supplementary Figure 1).

### Segregation analysis

Sanger sequencing confirmed the hemizygous mutation in the proband and identified his mother as a heterozygous carrier (Figure 4B). The proband’s father and sister were wild-type at this position (Figure 4C). The mutation was absent in 100 ethnically matched control subjects and has not been reported in population databases.

### *In Silico* pathogenicity prediction

All computational prediction algorithms uniformly indicated pathogenicity:

- PolyPhen-2: Probably damaging (score: 1.000)
- SIFT: Deleterious (score: 0.000)
- MutationTaster: Disease-causing (probability: >0.999)
- CADD: 32.0 (indicating top 0.1% most deleterious variants)
- REVEL: 0.962 (strongly pathogenic)

The mutation introduces significant physicochemical changes: replacement of hydrophilic serine with hydrophobic phenylalanine at position 47 increases local hydrophobicity index from −0.8 to 2.8 (Kyte-Doolittle scale) and side chain volume from 89 Ų to 189 Ų, potentially disrupting local protein folding.

The variant was classified as Likely Pathogenic according to ACMG/AMP guidelines (criteria: PM2 - absent from population databases including gnomAD and 1,000 Genomes; PM4 - in-frame deletion/insertion in a non-repetitive region without length change; PP3 - multiple computational tools (PolyPhen-2, SIFT, MutationTaster, CADD, REVEL) predict deleterious effects; PP4 - patient’s phenotype is highly specific for NDP-associated Norrie disease).

### Clinical outcome

The patient underwent penetrating keratoplasty for the right eye due to persistent corneal edema and pain from acute hydrops. Postoperative examination revealed retinal pathology consistent with Norrie disease, similar to the left eye findings. At six-month follow-up, the corneal graft remained clear, but visual acuity showed no improvement due to underlying retinal pathology.

## Discussion

This study presents the first documented case of Norrie disease manifesting with acute keratoconus as the presenting clinical feature, caused by a novel *NDP* mutation (p. Ser47_Ile48delinsPheLeu). This unique presentation expands the recognized phenotypic spectrum of Norrie disease and highlights the importance of comprehensive genetic evaluation in patients with complex ocular abnormalities.

The novel mutation identified in our patient represents a complex indel affecting two adjacent amino acids immediately upstream of the cysteine knot domain. The cysteine knot, characterized by three conserved disulfide bonds (Cys55-Cys110, Cys69-Cys126, and Cys70-Cys127), forms the structural scaffold essential for Norrin protein stability and function (9). The substitution of serine with phenylalanine at position 47 introduces a bulky hydrophobic residue that likely disrupts the local secondary structure and may interfere with proper folding of the adjacent cysteine knot domain. Previous crystallographic studies have shown that even minor perturbations in this region can abolish Norrin-FZD4 interaction (5).

Given that Norrie disease classically affects posterior segment development (retinal vascularization and inner ear), the prominent anterior segment abnormalities (keratoconus with acute hydrops, spherophakia, and shallow anterior chambers) in our patient are unusual. To explore potential mechanisms, we considered NDP expression patterns. GTEx data show low NDP expression in most non-ocular adult tissues (highest in brain regions, minimal in connective tissues), but GTEx lacks eye-specific samples such as cornea, lens, or anterior segment structures, limiting its utility for assessing ocular expression. However, targeted studies have demonstrated NDP/Norrin expression in developing ocular tissues, particularly in Müller glia and retinal/choroidal endothelial cells, where it regulates vascular development via Wnt/*β*-catenin signaling and cross-inhibition with TGF-β pathways (10), While direct evidence of Norrin expression in corneal stroma or keratocytes remains limited, these findings raise the possibility that certain NDP variants—especially those causing partial loss-of-function near the cysteine knot—may exert tissue-specific or developmental-stage-specific effects on anterior segment structures, potentially through indirect modulation of extracellular matrix integrity or stromal cell signaling during embryogenesis.

The association between Norrie disease and keratoconus has not been previously reported. A 1991 report described ‘keratotorus’ (likely keratoconus) in a 46-year-old with advanced Norrie disease (11), but not as the initial manifestation. Our case is unique in presenting with acute hydrops leading to diagnosis. While corneal abnormalities in Norrie disease typically manifest as late-stage complications secondary to chronic angle-closure glaucoma or phthisis bulbi (12), our patient presented with primary corneal ectasia. Several hypotheses may explain this unusual phenotype:

First, the specific mutation location may confer a unique functional consequence. Unlike most reported *NDP* mutations affecting the cysteine knot domain itself, our mutation affects residues immediately adjacent to this critical region, potentially resulting in a partial loss-of-function with tissue-specific effects (8).

Second, the concurrent presence of spherophakia and shallow anterior chambers in our patient suggests a broader anterior segment developmental abnormality. Recent studies have identified Norrin expression in developing anterior segment structures, though its precise role remains unclear (10). The combination of spherophakia and keratoconus in our patient may represent a previously unrecognized anterior segment phenotype associated with specific *NDP* mutations. This is supported by evidence of Norrin expression in developing anterior segment structures, where it modulates TGF-*β* signaling to influence vasculature and potentially stromal integrity (10). A hypothesis for keratoconus development is that disrupted Norrin/Wnt signaling in corneal fibroblasts leads to extracellular matrix dysregulation, promoting ectasia—analogous to Wnt’s role in corneal wound healing and biomechanics.

Third, genetic modifiers or environmental factors may contribute to the keratoconus phenotype. Several genes involved in corneal biomechanical properties, including COL5A1, LOX, and ZNF469, have been associated with keratoconus susceptibility (13). These could interact with NDP dysfunction, amplifying anterior segment effects in susceptible individuals. While our whole-genome sequencing did not identify obvious modifiers, future studies sequencing these loci in Norrie cohorts could clarify interactions. Investigation of potential genetic modifiers in our patient may provide insights into the phenotypic variability observed in Norrie disease.

The preservation of normal intraocular pressure in our patient, despite significant anterior segment abnormalities, distinguishes this presentation from typical secondary glaucoma seen in advanced Norrie disease. The absence of optic disc cupping and retinal nerve fiber layer thinning on OCT further supports the primary nature of the corneal pathology rather than a secondary glaucomatous process.

From a clinical management perspective, this case underscores several important considerations. First, Norrie disease should be considered in the differential diagnosis of males presenting with bilateral keratoconus and additional ocular abnormalities, particularly when associated with poor vision from early childhood. Second, comprehensive genetic testing can provide definitive diagnosis and guide appropriate genetic counseling for affected families. Third, while surgical intervention for acute hydrops may address immediate symptoms, the underlying retinal pathology limits visual rehabilitation potential.

### Study limitations

This study has several limitations. First, functional characterization of the novel mutation through *in vitro* expression studies was not performed. Second, we did not conduct whole-exome or whole-genome sequencing on family members to investigate potential genetic modifiers. Third, auditory and neurodevelopmental assessments were not systematically performed, limiting our ability to fully characterize the systemic phenotype.

### Clinical implications and future directions

The identification of keratoconus as a potential manifestation of Norrie disease has important implications for clinical practice and genetic counseling. Ophthalmologists should maintain a high index of suspicion for underlying genetic disorders in patients presenting with bilateral keratoconus and additional ocular abnormalities. Early genetic diagnosis enables appropriate family counseling regarding recurrence risks and carrier testing.

Future studies should investigate the prevalence of corneal ectatic disorders in larger cohorts of Norrie disease patients and explore potential genotype–phenotype correlations. Functional characterization of mutations affecting regions adjacent to the cysteine knot domain may provide insights into structure–function relationships and tissue-specific effects of different *NDP* variants.

## Conclusion

We report a novel NDP mutation (p. Ser47_Ile48delinsPheLeu) in a Chinese family with Norrie disease, where acute keratoconus with hydrops served as the initial diagnostic manifestation, expanding the phenotypic spectrum. This finding significantly expands the clinical phenotypic spectrum of Norrie disease and enriches the *NDP* mutational database. Our study emphasizes the importance of genetic testing in patients with complex ocular phenotypes and provides valuable information for diagnosis, prognosis, and genetic counseling of affected families.

## Data Availability

The original contributions presented in the study are included in the article/Supplementary material, further inquiries can be directed to the corresponding author.
