# Supplementary material for: Case Report: Acute keratoconus as the presenting feature in undiagnosed Norrie disease: hypothesis from a novel NDP mutation
Source: Front Med (Lausanne). 2026 Mar 30;13:1726644. doi: 10.3389/fmed.2026.1726644 (PMC13070777; doi:10.3389/fmed.2026.1726644)
Supplement: Supplementary file 1 [file Supplementary_File_1.docx]

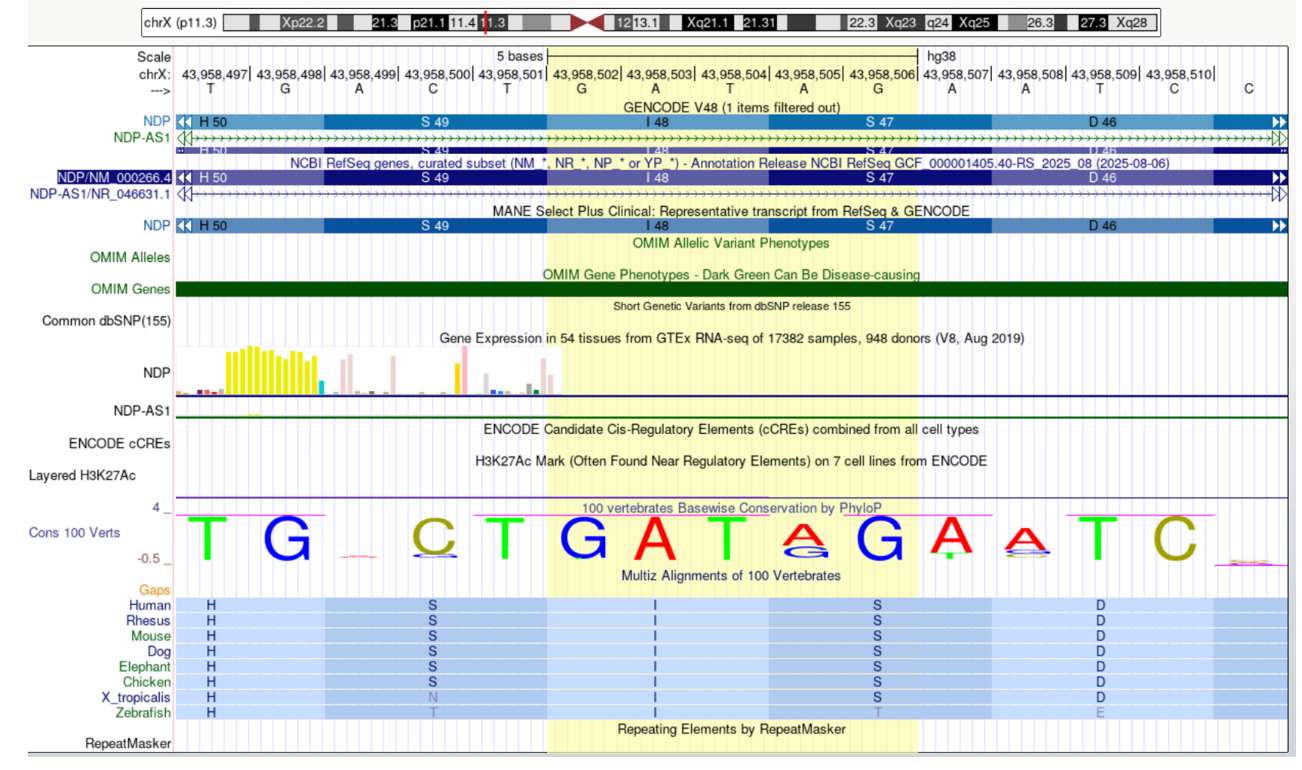


**Supplementary Figure 1.** Conservation analysis of the NDP gene mutation site using UCSC Genome Browser. The figure displays the genomic context of the c.140_144delinsTTTTA mutation (red arrow) in the NDP gene on chromosome X (chrX:43,958,497-43,958,501, GRCh38/hg38). The mutation site corresponds to amino acids Ser47-Ile48 (S-I) in the protein sequence. The bottom panel shows evolutionary conservation across 100 vertebrate species, demonstrating complete conservation of both serine (S) and isoleucine (I) residues at positions 47 and 48 across all examined species including human, rhesus, mouse, dog, elephant, chicken, *Xenopus tropicalis*, and zebrafish. The conservation track (Cons 100 Verts) indicates high PhyloP conservation scores at the mutation site. Additional tracks show GENCODE gene annotation, OMIM disease associations (green bar indicating disease-causing variants), gene expression data from GTEx, and regulatory elements. The yellow-highlighted regions indicate areas of high conservation and regulatory importance. This complete conservation across diverse vertebrate species spanning over 400 million years of evolution strongly supports the pathogenic nature of the identified mutation.
